# 

**Published:** 1884-07-20

**Authors:** 


### TABLE OF CONTENTS.

ORIGINAL AND SELECTED ARTICLES:

Hypodermic use of Carbolic Acid for the Cure of Erysipelas, by J. McF. Gaston, M.D., Atlanta. Ga., 241. Bladder Worms, By E. H. M. Parham, M D, of Arkansas, 244. The Constipation Habit, 245. Local Anæsthesia, 248. How to Secure Good Dental Organs, and Preserve them from Decay, Prevent Rickets, Hip Disease. etc., 249. Review of the Progress of Medical and Surgical Electricity, 251. Diarrhœa in Children, 253. A Case of Conception without Intromission, 256. On Reduction of Dislocations by Manipulation, 257. The Law Case of Drs. Bower and Keates, 258.

ABSTRACTS AND GLEANINGS:

Retained Placenta, 259. Calomel an Instantaneous Nervous Sedative, 260. Caffein in Therapeutic Doses; Placenta Prævia, 261. The Hypodermic Injection of Piles: Exit National Board of Health, 262 The Cholera Bacillus; Cannabis Indica, 263 Chronic Dyipepsia, 264. Carbolic Acid Injections of Piles; Alumen Ustum in Intermittent Fever; Chloroform Narcosis During Sleep, 265. Salicylic Acid a Cure for Tic Douloureux; Sulphide of Calcium for Scabies; Extirpation of Goitre by means of the Elastic Ligature, 266. Treatment of Intermittent Fever; Drugs Influencing the Sexual Powers, 267. Bromides in Treatment of Neurotic Diarrhœa of Children; Too Much Feeding of Infants, 268. Humane Blistering; Varicose Veins; Bursting of Coffin, 269. Etherization by Rectum; Cod Liver Oil as a Surgical Dressing; Eucalyptus; Iodine in Intermittent Fever; Substitute for Opium, 270.

SCIENTIFIC ITEMS:

Victor Hugo on Sewage Irrigation, 271. Ericsson's New Solar Motor; Dreams, 272.

PRACTICAL NOTES AND FORMULÆ.

Sadberry Spleen Mixture; Remedy for Neuralgic Dysmenorrhœa; Soldier's Itch, 273. Remedy for Weakness of the Sexual Organs; Prevention of Abscess: Syrup of Codeia.—For Coughs; Treatment of Infantile Eclampsia, 274.

EDITORIALS AND MISCELLANEOUS.

Editorial Notices, 275. Southern Medical College; Cholera, 276. Book Notices, 277. Receipted; Special Notices, 280.

